# First-in-human phase I open-label study of the LAG-3 antagonist antibody INCAGN02385 in patients with select advanced or metastatic solid tumors

**DOI:** 10.1093/oncolo/oyaf136

**Published:** 2025-07-09

**Authors:** John D Powderly, Martin E Gutierrez, Ani S Balmanoukian, Paul E Hoyle, Zhiwan Dong, Lulu Cheng, Xuejun Chen, John E Janik, Nawel Bourayou, Omid Hamid

**Affiliations:** Carolina BioOncology Institute, Huntersville, NC 28078, United States; Hackensack University Medical Center, Hackensack, NJ 07601, United States; The Angeles Clinic and Research Institute, a Cedars Sinai Affiliate, Los Angeles, CA 90025, United States; Incyte Corporation, Wilmington, DE 19803, United States; Incyte Corporation, Wilmington, DE 19803, United States; Incyte Corporation, Wilmington, DE 19803, United States; Incyte Corporation, Wilmington, DE 19803, United States; Incyte Corporation, Wilmington, DE 19803, United States; Incyte Biosciences International Sàrl, 1110 Morges, Switzerland; The Angeles Clinic and Research Institute, a Cedars Sinai Affiliate, Los Angeles, CA 90025, United States

**Keywords:** immune checkpoint inhibitors, monoclonal antibodies, carcinoma, metastases, immunotherapy, clinical trial phase I

## Abstract

**Background:**

Immune checkpoint receptor lymphocyte-activation gene 3 (LAG-3) is an activation marker for CD4^+^ and CD8^+^ T cells. Prolonged LAG-3 expression downregulates T-cell activation; therefore, LAG-3 blockade may restore antitumor immune response. INCAGN02385 is a humanized monoclonal LAG-3-targeting antibody. This first-in-human phase I study evaluated INCAGN02385 for advanced/metastatic solid tumors.

**Materials and Methods:**

In this dose escalation study, patients with select immunogenic advanced or metastatic solid tumors received a single INCAGN02385 infusion (25 mg to 750 mg) every 2 weeks (Q2W). Objectives included evaluation of safety/tolerability, maximum tolerated dose (MTD) (primary), pharmacokinetics (PK), antitumor activity (secondary).

**Results:**

Twenty-two patients were enrolled and treated. Sixty-four percent had received ≥ 3 lines of systemic therapy. Sixty-eight percent had received prior immune checkpoint inhibitor (ICI) therapy; anti–programmed death protein-1/anti–programmed death ligand-1, 68%, anti–cytotoxic T-lymphocyte-associated protein-4 therapy, 18%. No dose-limiting toxicities occurred, and an MTD was not reached. Sixteen patients (73%) experienced treatment-related adverse events (TRAEs), most frequently fatigue (*n* = 7). Except for one grade 3 lymphopenia TRAE, all were grade 1/2 severity. Two patients experienced sponsor-assessed immune-related AEs (pneumonitis, peripheral sensory neuropathy [*n* = 1] patient each). INCAGN02385 PK parameters were dose proportional across all doses evaluated. Six patients achieved stable disease lasting ≥ 56 days (range, 57-413 days).

**Conclusions:**

INCAGN02385 exhibited linear PK and preliminary evidence of disease control in this heavily pretreated population, consistent with other LAG-3-targeting monotherapies. A 350-mg Q2W dose was selected for phase II studies that will focus on combinations of INCAGN02385 with other ICIs.

**Implications for Practice**

Despite substantially positive impacts of PD-1/PD-L1 blockade on cancer treatment and patient outcomes, unmet needs remain, owing to low response rates in some tumors and treatment resistance in some patients. Targeting additional checkpoints may in part overcome these unmet needs. Prolonged LAG-3 expression downregulates T-cell activation; therefore, LAG-3 blockade may restore antitumor immune response, representing a potential novel treatment. Anti–LAG-3 antibody INCAGN02385 was generally well tolerated and showed preliminary modest disease control in this phase I study, consistent with other LAG-3-targeting monotherapies. Phase II studies will investigate INCAGN02385 combinations with other ICIs, where LAG-3 has been validated as a target.

## Introduction

Programmed death protein-1 (PD-1)/programmed death ligand-1 (PD-L1) blockade has revolutionized the treatment of cancer^1^; however, although several tumor types are responsive to PD-1-/PD-L1-targeted monotherapy, response rates are low in multiple tumor types and many patients that do respond subsequently acquire resistance.^2-8^ The discovery of novel immune checkpoints in addition to PD-1/PD-L1 has prompted interest in investigating whether agents designed to inhibit these additional checkpoints as single agents or in combination might address the unmet need for enhanced antitumor effects.^8-10^

The immune checkpoint receptor lymphocyte-activation gene 3 (LAG-3) is an activation marker for CD4^+^ and CD8^+^ T cells and other lymphocytes.^11-14^ As observed with other immune-modulating receptors, such as PD-1, prolonged expression of LAG-3 negatively regulates T-cell activation and function, leading to T-cell exhaustion and tumor immune escape.^11-13,15^ Therefore, blockade of LAG-3 in the tumor microenvironment may restore antitumor immune response. In preclinical murine models, combined targeting of LAG-3 and PD-1 reduced tumor growth compared with PD-1-targeted monotherapy,^16^ and combined anti–LAG-3 plus anti–PD-L1 therapy improved survival vs anti–PD-L1 therapy alone.^17^ Several LAG-3 inhibitors, including the monoclonal antibodies (mAbs) favezelimab,^18^ ieramilimab,^19^ and relatlimab,^20^ have also been investigated in clinical trials as monotherapy and in combination with other immune checkpoint inhibitors (ICIs), including the anti–PD-1 mAbs pembrolizumab, spartalizumab, and nivolumab, for treatment of various malignancies. In 2022, the US Food and Drug Administration approved a fixed-dose combination of nivolumab and relatlimab for unresectable or metastatic melanoma,^21^ in which this combination therapy prolonged median progression-free survival (PFS) relative to nivolumab alone (10.1 vs 4.6 months) in previously untreated patients.^20^ Median PFS estimates were longer for relatlimab plus nivolumab vs nivolumab alone (12.6 vs 4.8 months) among patients with LAG-3 expression levels ≥ 1% of all nucleated tumor cells but were similar among those with PD-L1 expressed by ≥ 1% (15.7 vs 14.7 months).^20^ A treatment benefit with relatlimab plus nivolumab compared with nivolumab alone was observed regardless of patients’ LAG-3 expression or *BRAF* mutation status.^20^

INCAGN02385 is a humanized Fc-engineered immunoglobulin G1κ (aglycosylated, N297A) mAb designed to specifically target the D1 domain of LAG-3. INCAGN02385 disrupts LAG-3–major histocompatibility complex class II binding, which enhances T-cell receptor signaling and cytokine secretion of activated T cells.^22,23^ In preclinical studies, INCAGN02385 demonstrated high affinity binding to human LAG-3, strong ability to increase the cytotoxicity of exhausted T cells, and enhanced antitumor activity in combination with an anti–PD-1 antibody in murine models vs anti–PD-1 monotherapy^23^ (data on file, Incyte Corporation). Results from a first-in-human study of safety and tolerability of INCAGN02385 in patients with advanced or metastatic solid tumors are reported herein.

## Materials and methods

### Study design

This phase I open-label nonrandomized study evaluated the safety, tolerability, pharmacokinetics (PK), and preliminary antitumor activity of INCAGN02385 in the treatment of patients with advanced or metastatic solid tumors (ClinicalTrials.gov identifier, NCT03538028). The study was conducted at 4 centers in the United States. INCAGN02385 dose was escalated using 5 dose levels (25, 75, 250, 350, and 750 mg; Figure S1) with infusions administered every 2 weeks (Q2W) on day 1 of each 14-day cycle. The study used a 3 + 3 dose escalation design to identify the maximum tolerated dose (MTD), the maximum number of tolerated doses (MNTD), or pharmacologically active dose (PAD) of INCAGN02385 in patients with select immunogenic tumor types. Approximately 3-6 patients were to be enrolled at each dose level. Dose escalation rules are presented in Supplementary Material. The maximum tolerated dose was defined as one dose level below that at which at least one-third of patients in a specific cohort have investigator-assessed dose-limiting toxicities (DLTs, monitored during a 28-day observation period) (Table S1).

### Ethics approval

The study was performed in accordance with the ethical principles of the International Council for Harmonisation of Technical Requirements for Pharmaceuticals for Human Use guideline for Good Clinical Practice, the Declaration of Helsinki, and other applicable local ethical and legal requirements. The protocol (including amendments) was approved by an independent ethics committee or institutional review board before enrollment of patients at each site (Sites 1-3, Western Institutional Review Board; Site 4, Vanderbilt University Institutional Review Board). All patients provided written informed consent before screening.

### Patients

Eligible patients were aged ≥ 18 years with either previously treated locally advanced or metastatic solid tumors not amenable to resection with curative intent, or metastatic disease, and disease progression following, or intolerance to, all available therapies. Tumor types eligible for inclusion in the study were immunogenic tumors for which PD-1/PD-L1 therapy has evidence of efficacy. Additional immunogenic tumor types were allowed subject to the sponsor’s approval. Eligible patients had measurable disease per Response Evaluation Criteria in Solid Tumors (RECIST) v1.1, had Eastern Cooperative Oncology Group performance status (ECOG PS) ≤ 1, and were required to provide pretreatment (fresh or archival) biopsies. On-treatment tumor biopsies were optional.

Exclusion criteria included the history of anti–LAG-3 antibody therapy (for any indication), as well as treatment with chemotherapy, targeted small molecule therapy, PD-1 pathway-targeted agents, or radiotherapy within 14 days, or mAb or other investigational agent, immune suppressive treatment, or receipt of a live vaccine within 28, 7, and 30 days, respectively, of the start of the study.

### Objectives and assessments

The primary objectives of the study were to assess the safety and tolerability of INCAGN02385. This included the identification of DLTs (Table S1), assessment of MTD (Supplementary Material) or PAD, and assessment of frequency, duration, and severity of treatment-emergent adverse events (TEAEs). Treatment-emergent adverse events were summarized using the Medical Dictionary for Regulatory Activities (MedDRA; version 23.1) preferred terms. TEAE severity was graded based on the National Cancer Institute Common Terminology Criteria for Adverse Events v4.03. Adverse events (AEs) with a potential immunologic etiology (immune-related AEs [irAEs]) were defined as AEs consistent with an immune phenomenon associated with drug exposure after all other etiologies were eliminated. These were identified by the sponsor based on predefined MedDRA preferred terms that are associated with symptoms arising from each irAE. Based on the clinical review by the sponsor, irAEs were assessed for immune relatedness.

Secondary objectives were to evaluate PK and preliminary antitumor activity of INCAGN02385. PK endpoints included maximum observed serum concentration (C_max_), time to maximum concentration (t_max_), and area under the serum concentration–time curve (AUC) from time 0 to the last measurable concentration at time t (AUC_0–t_). Antitumor activity endpoints included objective response rate (ORR), duration of response (DOR), PFS, and disease control rate. Objective response rate, DOR, and PFS were based on the investigator assessment of radiographic disease (per RECIST v1.1).^24^

Exploratory objectives included assessment of INCAGN02385 immunogenicity (defined as the occurrence of specific antidrug antibodies [ADAs] to INCAGN02385), association of INCAGN02385 PK with receptor occupancy, and investigation of tumor and immune cell infiltrate biomarkers potentially predictive of INCAGN02385 pharmacological activity.

### Pharmacokinetic and ADA analyses

Serum samples were collected preinfusion on day 1 of cycles 1, 2, 3, 6, 8, and 12 (PK and ADA analyses), and preinfusion on day 1 of cycles 4 and 7 (PK analysis). Serum samples were also collected postinfusion on day 1 of cycles 1 and 6, 4 hours postinfusion on day 1 of cycles 1 and 6, and 24 hours postinfusion on day 2 of cycles 1 and 6 (PK analysis). Untimed samples were also collected on day 8 of cycles 1 and 6 (PK analysis) and at the first safety follow-up visit (PK and ADA analyses).

An enzyme-linked immunosorbent assay developed and validated by Frontage Laboratories was used to quantify INCAGN02385 in serum samples (Supplementary Material).

### Tumor imaging

Tumor imaging was performed using contrast computed tomography (CT) or magnetic resonance imaging (or CT component of a positron emission tomography/CT with the sponsor’s approval). Chest and abdomen images were required for all patients. Imaging was performed at screening, 8 weeks after the first dose of INCAGN02385, then every 8 weeks for 12 months, and every 12 weeks thereafter until disease progression was determined. Response to INCAGN02385, per modified RECIST v1.1, was confirmed by repeat radiographic assessment ≥ 4 weeks from the date of first documented response or at the next scheduled scan, whichever was clinically indicated. Patients who showed radiologic evidence of disease progression, per RECIST v1.1, could continue treatment ahead of investigator-assessed immune RECIST (iRECIST) confirmation by repeat imaging > 4 and ≤ 8 weeks after initial indication, if they were considered clinically stable per investigator assessment. Clinical stability was defined as the absence of any symptoms and signs of clinically significant disease progression, no decline in ECOG PS, and no need for intensified management, including increased analgesia, radiation, or palliative care. Repeat imaging was not required for patients not deemed clinically stable; these patients were discontinued following central verification of initial radiological evidence of disease progression.

### Correlative translational studies

#### LAG-3 receptor occupancy analysis

LAG-3 receptor occupancy was determined using flow cytometry to detect total and free LAG-3 on activated T cells (from healthy donors) incubated with patient serum samples taken before and after treatment with INCAGN02385. Receptor occupancy in peripheral blood was calculated as 100 × (1-free LAG-3 mean fluorescent intensity [MFI]/total LAG-3 MFI) (Supplementary Material).

#### Plasma protein biomarker analysis and whole blood T-cell population profiling

Immune and nonimmune plasma proteins were identified using a multiplex proximity extension assay performed by Olink Proteomics. Frequencies of immune cells in the peripheral blood of patients were monitored using multicolor flow cytometry performed by Caprion Biosciences. Two flow cytometry panels were used to evaluate T-cell subsets (memory/naive and regulatory T [Treg]) and T-cell function (activation/exhaustion). The marker for T-cell proliferation was Ki67, and the markers for Treg cells were CD3^+^, CD4^+^, CD25^+^, CD127^−^, and FoxP3^+^.

### Statistical analysis

The full analysis set included all patients enrolled in the study who received ≥ 1 dose of INCAGN02385 and was used to analyze demographic and baseline characteristics, safety data, and study drug administration and efficacy data. The PK-evaluable population included all patients who received ≥ 1 dose of INCAGN02385 and had ≥ 1 postdose PK sample collected and analyzed. The translational pharmacodynamic–evaluable population included all patients who received ≥ 1 dose of INCAGN02385 and had ≥ 1 postdose translational pharmacodynamic blood sample collected and analyzed. Treatment-emergent adverse events and laboratory values were summarized with descriptive statistics. No formal statistical tests were performed in this exploratory study, and all CIs were reported to 95% confidence levels.

Objective response rates and 95% CIs were determined for the RECIST-evaluable population and presented by treatment group. Progression-free survival was analyzed by the Kaplan-Meier method, and median PFS was estimated with 95% CIs for each treatment group. Effects of treatment on immune cell frequencies were evaluated using the paired *t*-test. Treatment effects on plasma protein biomarkers were evaluated by paired least squares mean *t*-test comparing baseline (cycle 1 day 1) with on-treatment (cycle 1 day 8 and/or cycle 2 day 1) values. Differences were considered significant at a false discovery rate < 0.05.

## Results

### Patients

A total of 22 patients were enrolled between June 18, 2018, and October 7, 2020. The date of database lock was February 5, 2021. Median (range) age was 63.0 (46-86) years, 55% of patients were male, and the majority were White (91%) (Table 1). All patients had an ECOG PS of either 0 (18%) or 1 (82%). The most frequent solid tumor type was lung cancer (*n* = 4 [18%]); 21 patients (95%) had metastatic disease at study entry, including metastases of the lymph nodes (*n* = 12 [55%]), lung (*n* = 8 [36%]), liver (*n* = 6 [27%]), and bone (*n* = 5 [23%]).

**Table 1.** Patient baseline demographics and characteristics.

Characteristic	INCAGN02385 treatment group					
	25 mg Q2W (*n* = 4)	75 mg Q2W (*n* = 4)	250 mg Q2W (*n* = 4)	350 mg Q2W (*n* = 3)	750 mg Q2W (*n* = 7)	Total (*N* = 22)
Age (years), median (range)	59.0 (46-61)	63.0 (46-74)	67.0 (58-86)	59.0 (53-77)	66.0 (49-74)	63.0 (46-86)
Sex, *n* (%) Male Female	1 (25) 3 (75)	1 (25) 3 (75)	3 (75) 1 (25)	2 (67) 1 (33)	5 (71) 2 (29)	12 (55) 10 (45)
Race, *n* (%) White Black Asian Other	4 (100) 0 0 0	3 (75) 0 1 (25) 0	3 (75) 0 0 1 (25)	3 (100) 0 0 0	7 (100) 0 0 0	20 (91) 0 1 (5) 1 (5)
Tumor type, *n* (%) Adenocarcinoma of the endometrium Breast cancer Gastric cancer Lung cancer Melanoma Ovarian cancer Other	0 1 (25) 0 2 (50) 0 0 1 (25)^a^	1 (25) 0 0 0 0 2 (50) 1 (25)^b^	0 0 0 2 (50) 0 0 2 (50)^c^	0 0 2 (67) 0 1 (33) 0 0	1 (14) 1 (14) 0 0 1 (14) 0 4 (57)^d^	2 (9) 2 (9) 2 (9) 4 (18) 2 (9) 2 (9) 8 (36)
Number of metastatic sites at study entry, *n* (%) <3 ≥3	1 (25) 3 (75)	1 (25) 3 (75)	1 (25) 3 (75)	1 (33) 2 (67)	5 (71) 2 (29)	9 (41) 13 (59)
ECOG PS, *n* (%) 0 1	0 4 (100)	1 (25) 3 (75)	1 (25) 3 (75)	0 3 (100)	2 (29) 5 (71)	4 (18) 18 (82)
Types of prior therapy, *n* (%) Surgery Radiotherapy Systemic therapy	2 (50) 3 (75) 4 (100)	4 (100) 4 (100) 4 (100)	4 (100) 0 4 (100)	2 (67) 1 (33) 3 (100)	5 (71) 5 (71) 7 (100)	17 (77) 13 (59) 22 (100)
Prior anticancer systemic therapy type, *n* (%) Chemotherapy Targeted therapy Checkpoint inhibitor therapy Other immunotherapy^e^	4 (100) 3 (75) 3 (75) 1 (25)	3 (75) 3 (75) 2 (50) 0	3 (75) 3 (75) 4 (100) 1 (25)	2 (67) 0 1 (33) 0	4 (57) 2 (29) 5 (71) 1 (14)	16 (73) 11 (50) 15 (68) 3 (14)
Number of lines of prior anticancer systemic therapy, *n* (%) 0-1 2 ≥3	0 0 4 (100)	0 1 (25) 3 (75)	0 0 4 (100)	2 (67) 1 (33) 0	2 (29) 2 (29) 3 (43)	4 (18) 4 (18) 14 (64)

Other solid tumors, all *n* = 1: ^a^Salivary gland cancer; ^b^Renal cell carcinoma; ^c^Bladder cancer, hepatocellular carcinoma; ^d^Basosquamous carcinoma, esophageal cancer, Merkel cell cancer, prostate cancer. ^e^All patients with a history of other immunotherapy also had a history of treatment with a checkpoint inhibitor.

Abbreviations: ECOG PS, Eastern Cooperative Oncology Group performance status; Q2W, every 2 weeks.

All patients had received prior anticancer systemic therapy and 64% had received ≥ 3 lines of therapy (Table 1). Most patients (15/22, 68%) had received prior ICI therapy; 15/22 (68%) had received anti–PD-1/PD-L1 therapy (most commonly nivolumab, *n* = 11), and 4 (18%) had received prior cytotoxic T-lymphocyte-associated protein-4 (CTLA-4) therapy (ipilimumab) in addition to PD-1 therapy. Sixteen patients (73%) had received prior chemotherapy, 17 (77%) had undergone prior surgery or procedure, and 13 (59%) had prior radiotherapy.

Patients received INCAGN02385 Q2W at doses of 25 mg, 75 mg, 250 mg (*n* = 4 each), 350 mg (*n* = 3), or 750 mg (*n *= 7). All patients discontinued treatment, most commonly due to progressive disease (*n* = 18 [82%]) (Figure S1). No on-treatment biopsies were collected.

### Safety and tolerability

Patients received a median (range) of 4.0 (1-26) INCAGN02385 Q2W infusion cycles, ranging from 2.5 (1-18) for the 250-mg cohort to 14 (5-15) for the 350-mg cohort. No patient experienced a DLT or investigator-assessed infusion-related reaction, and MTD and MNTD were not reached. All patients experienced ≥ 1 TEAE, most commonly fatigue (*n* = 8 [36%]), cough (*n* = 6 [27%]), and hypokalemia, nausea, and tumor pain (each *n* = 5 [23%]) (Table 2). Fourteen patients (64%) experienced ≥ 1 grade 3 TEAE (Table 2). Overall, grade 3 events occurred most commonly in patients receiving 750 mg Q2W (*n* = 5), and 25 mg and 75 mg Q2W (each *n* = 3) (Table 2). Sixteen patients (73%) experienced ≥ 1 treatment-related TEAE (TRAE), most frequently fatigue (*n* = 7 [32%]), increased blood creatinine, lymphopenia, myalgia, pruritus, and tumor pain (each *n* = 2 [9%]) (Table S2). Most events were grade 1/2; 1 patient (5%) experienced grade 3 lymphopenia. One patient (5%) experienced a TEAE (transient ischemic attack, not considered treatment-related by investigators) leading to INCAGN02385 discontinuation, and 6 (27%) experienced ≥ 1 TEAE leading to dose interruption, with only one event (increased blood creatinine event in a patient receiving 250 mg Q2W) considered treatment-related by investigators.

**Table 2.** Treatment-emergent adverse events.^a^

MedDRA preferred term, *n* (%)	INCAGN02385 treatment group					
	25 mg Q2W (*n *= 4)	75 mg Q2W (*n *= 4)	250 mg Q2W (*n *= 4)	350 mg Q2W (*n *= 3)	750 mg Q2W (*n *= 7)	Total (*N* = 22)
Any-grade TEAEs	4 (100)	4 (100)	4 (100)	3 (100)	7 (100)	22 (100)
Fatigue	2 (50)	1 (25)	0	3 (100)	2 (29)	8 (36)
Cough	2 (50)	1 (25)	2 (50)	0	1 (14)	6 (27)
Hypokalemia	1 (25)	0	0	3 (100)	1 (14)	5 (23)
Nausea	2 (50)	1 (25)	0	1 (33)	1 (14)	5 (23)
Tumor pain	2 (50)	2 (25)	0	1 (33)	0	5 (23)
Anemia	1 (25)	0	0	0	3 (43)	4 (18)
Constipation	0	2 (50)	0	1 (33)	1 (14)	4 (18)
Dyspnea	1 (25)	1 (25)	0	1 (33)	1 (14)	4 (18)
Hyponatremia	0	0	0	1 (33)	3 (43)	4 (18)
Pain in extremity	2 (50)	0	0	1 (33)	1 (14)	4 (18)
Vomiting	2 (50)	1 (25)	0	1 (33)	0	4 (18)
Activated partial thromboplastin time prolonged	0	0	1 (25)	1 (33)	1 (14)	3 (14)
Arthralgia	0	0	1 (25)	1 (33)	1 (14)	3 (14)
Blood creatinine increased	0	1 (25)	1 (25)	0	1 (14)	3 (14)
Headache	0	0	0	1 (33)	2 (29)	3 (14)
Hyperuricemia	0	0	0	1 (33)	2 (29)	3 (14)
Proteinuria	0	0	1 (25)	1 (33)	1 (14)	3 (14)
Grade ≥ 3 TEAE	3 (75)	3 (75)	2 (50)	1 (33)	5 (71)	14 (64)
Abdominal pain upper	0	0	1 (25)	0	0	1 (5)
Alanine aminotransferase increased	0	0	0	0	1 (14)	1 (5)
Bacteremia	0	0	0	0	1 (14)	1 (5)
Dehydration	0	0	0	0	1 (14)	1 (5)
Failure to thrive	0	0	1 (25)	0	0	1 (5)
Herpes zoster	1 (25)	0	0	0	0	1 (5)
Hip fracture	0	0	0	1 (33)	0	1 (5)
Hypercalcemia	0	0	0	0	1 (14)	1 (5)
Hyperglycemia	0	1 (25)	0	0	0	1 (5)
Hyponatremia	0	0	0	0	1 (14)	1 (5)
Leukocytosis	0	0	0	0	1 (14)	1 (5)
Lymphopenia	0	1 (25)	0	0	0	1 (5)
Malignant pleural effusion	1 (25)	0	0	0	0	1 (5)
Neuropathy peripheral	1 (25)	0	0	0	0	1 (5)
Neutrophil count decreased	0	0	0	0	1 (14)	1 (5)
Peripheral artery thrombosis	0	0	0	0	1 (14)	1 (5)
Peripheral ischemia	0	0	0	0	1 (14)	1 (5)
Pneumonia aspiration	0	0	1 (25)	0	0	1 (5)
Small intestinal obstruction	0	1 (25)	0	0	0	1 (5)
Decreased white blood cell count	0	0	0	0	1 (14)	1 (5)

^a^TEAEs occurring in ≥ 10% (any grade). One TEAE above grade 3 was reported during the study; failure to thrive grade 5 was reported for one patient in the 250 mg Q2W group.

Abbreviations: MedDRA, Medical Dictionary for Regulatory Activities; Q2W, every 2 weeks; TEAE, treatment-emergent adverse event.

Eight patients (36%) experienced ≥ 1 serious TEAE (acute kidney injury, bacteremia, dehydration, failure to thrive, herpes zoster infection, hip fracture, malignant pleural effusion, peripheral artery thrombosis, pneumonia aspiration, small intestinal obstruction, *n* = 1 each); none were considered treatment-related by investigators. Two patients experienced a sponsor-assessed irAE (pneumonitis, peripheral sensory neuropathy, *n* = 1 each), both were grade 1 and not considered related to INCAGN02385 by the investigators. One patient (5%) experienced a TEAE with a fatal outcome (failure to thrive due to progression of underlying disease in a patient receiving 250 mg Q2W), which was not considered treatment-related by investigators.

### Pharmacokinetics

**Figure 1.**
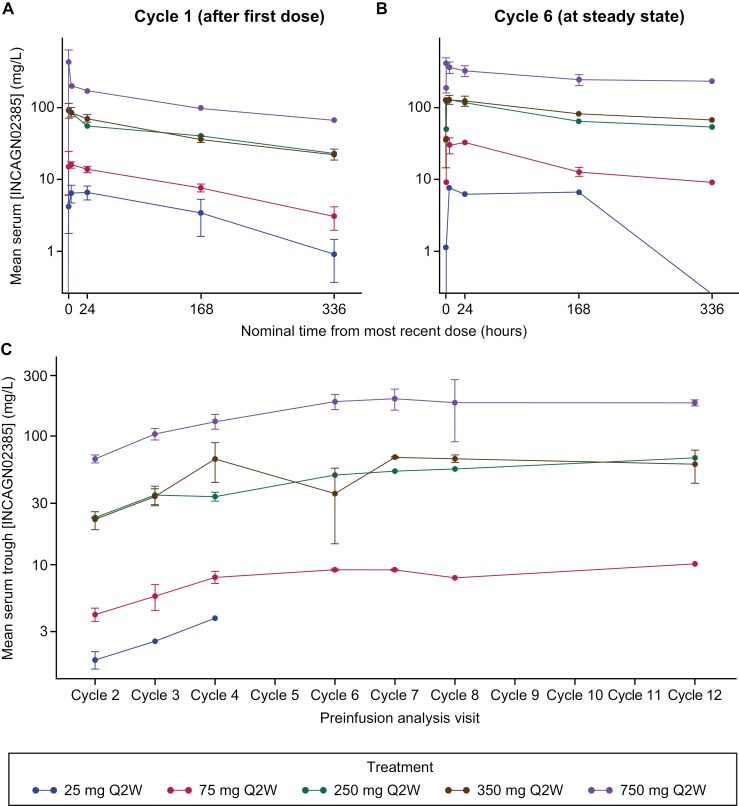
Mean (± SE) INCAGN02385 serum concentration over time by treatment group after (A) the first dose (cycle 1), (B) at steady state (cycle 6), and (C) serum preinfusion trough concentrations (C_min_) from cycle 2 to cycle 12. Trough concentrations below the lower limit of quantitation (< 1 mg/L) are excluded. C_min_, minimum observed serum concentration; Q2W, every 2 weeks.

INCAGN02385 serum concentration−time profiles after first dose (cycle 1) and at steady state (cycle 6) stratified by dose are presented in Figure 1; corresponding PK parameters are summarized in Table S3. INCAGN02385 C_max_, AUC_0–t_, and area under the serum concentration–time curve from time 0 to infinity (AUC_0–∞_) were dose proportional across all doses evaluated (Table S3, Figure S2). Dose proportionality was determined using the first-dose PK parameters of INCAGN02385 across all dose levels. The exponent values of the power function were 1.1 (90% CI, 1.0-1.2) for C_max_, 1.2 (90% CI, 1.0-1.4) for AUC_0–t_, and 1.1 (90% CI, 0.9-1.3) for AUC_0–∞_, which were not significantly different from 1.

At 350 mg Q2W, after the first dose, PK data were available for 3 patients; geometric mean C_max_, AUC_0–t_, and terminal half-life were 91 mg/L, 13 700 mg**·**h/L, and 173 hours. Median t_max_ for INCAGN02385 after the first dose was 4.1 hours (range, 0.5-4.7 hours). At steady state, PK data were available for 2 patients; individual geometric mean C_max_, AUC from time 0 to 336 hours postdose (AUC_0–336h_), and terminal half-life were 147 and 123 mg/L, 31 300 and 27 700 mg·h/L, and 121 and 349 hours, respectively. Individual patient values for t_max_ were 0.6 and 4.1 hours.

Clinical immunogenicity was assessed in 19 patients (19/22, 86%). Most patients (10/13) in dose groups with dose levels ≤ 350 mg Q2W were positive for treatment-emergent ADAs. None of the 6 patients in the 750 mg Q2W group were positive for treatment-emergent ADAs (Table S4). In 2 patients at the 25 mg Q2W dose level, preinfusion INCAGN02385 concentrations were below the lower limit of quantitation (< 1 μg/mL) at cycle 2 day 1 and all later cycles, likely due to the impact of ADAs. No impact on PK was seen at higher INCAGN02385 dose levels, based on increasing serum trough concentrations following multiple dose cycles; however, caution is needed in the interpretation of these data due to the limited number of PK observations at subsequent cycles.

### Correlative translational studies

**Figure 2.**
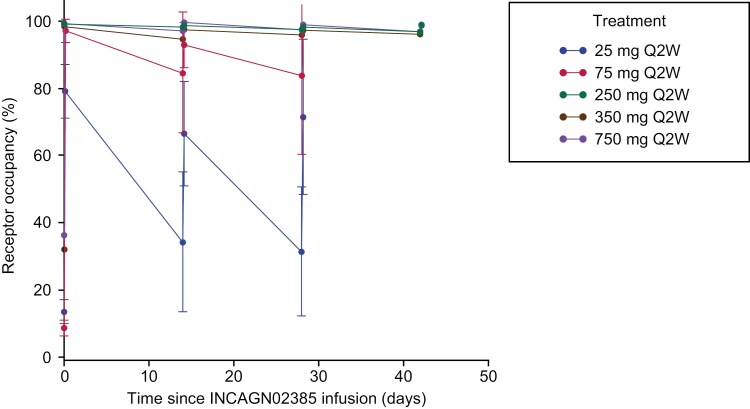
INCAGN02385 LAG-3 receptor occupancy. Average receptor occupancy (± SD) by treatment group at different time points post INCAGN02385 infusion. Number of patients with assessable data: 25 mg, 75 mg, and 750 mg, each *n* = 4; 250 mg, *n* = 2; 350 mg, *n* = 3. LAG-3, lymphocyte-activation gene 3; Q2W, every 2 weeks.

INCAGN02385 receptor occupancy on donor T cells was assessed in an in vitro assay with samples from 17 patients (72% of the total cohort). Mean maximum receptor occupancy values postdose (cycle 1 day 1) were 79% (25 mg), 97% (75 mg), 99% (250 mg), 98% (350 mg), and 99% (750 mg) (Figure 2). Full peripheral blood receptor occupancy was achieved at cycle 2 day 1 and at all subsequent time points when INCAGN02385 was administered at dose levels ≥ 250 mg. Trough receptor occupancy values at predose cycle 2 day 1 were 34% (25 mg), 85% (75 mg), 98% (250 mg), 95% (350 mg), and 97% (750 mg) (Figure 2).

**Figure 3.**
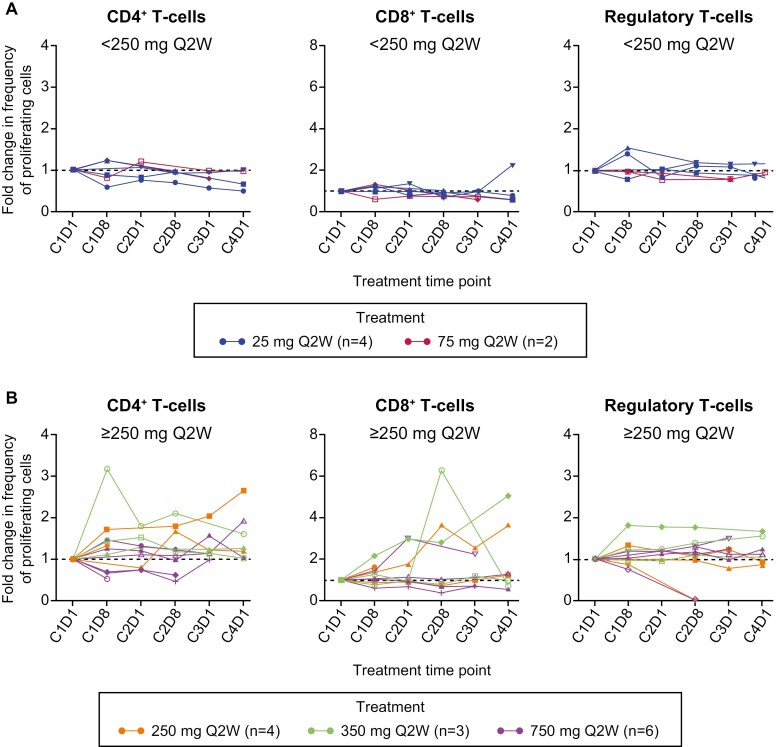
Changes in frequency of proliferating CD4^+^, CD8^+^, and regulatory T-cell activity in patients treated with INCAGN02385. Changes were normalized to the baseline level, and each individual patient is depicted by a single line. (A) INCAGN02385 dose levels < 250 mg: blue, 25 mg (*n* = 4); red, 75 mg (*n* = 2). (B) INCAGN02385 dose levels ≥ 250 mg: orange, 250 mg (*n* = 4); green, 350 mg (*n* = 3); purple, 750 mg (*n* = 6).

A subset of patients receiving INCAGN02385 dose levels ≥ 250 mg Q2W demonstrated an increase in the frequency of proliferating CD4^+^ T cells, which was significant at cycle 2 day 8 (*P* < .01). Although not significant, 3 patients receiving dose levels ≥ 250 mg Q2W demonstrated a sustained > 1-fold increase in proliferating CD8^+^ T cells. For these patients, the elevation was sustained at all subsequent time points for which data were available (up to cycle 3, *n* = 1; cycle 4, *n* = 2). No changes were observed in CD4^+^ or CD8^+^ T-cell proliferation in patients receiving doses of INCAGN02385 < 250 mg, or in Treg cells in patients receiving any dose (Figure 3A and B). No changes were observed in the frequency of coinhibitory receptor molecules PD-1, CTLA-4, T-cell immunoglobulin and mucin domain-containing protein-3 (TIM-3), and CD244 following treatment with INCAGN02385 (data on file, Incyte Corporation). Soluble LAG-3 in plasma was significantly elevated from baseline at cycle 1 day 8, with all doses of INCAGN02385 (false discovery rate < 0.05) (Figure S3A), suggesting LAG-3 receptor stabilization by the antibody; this elevation was not dose-dependent. No changes were observed in levels of interferon (IFN)γ-inducible proteins CXCL9, CXCL10, or CXCL11 (Figure S3B).

### Efficacy

Based on RECIST v1.1, no responders were identified. Across doses, 6 patients (27%; 95% CI, 11-50) achieved stable disease (SD) for ≥ 56 days (duration from the start of treatment until criteria for disease progression are met; range, 57-413 days; SD was ongoing at the time of censoring for patients reporting SD of 57 days and 413 days). Patients achieving SD had lung cancer, salivary gland cancer, hepatocellular carcinoma, gastric cancer, adenocarcinoma of the endometrium, or basosquamous carcinoma (all *n* = 1). All patients achieving SD had a history of progressive disease on prior therapies and all but one had a history of ICI treatment (Table S5). Median PFS across all patients was 1.9 months (95% CI, 1.9-2.5) (Table 3).

**Table 3.** Summary of best overall response, objective response rate, and disease control rate by RECIST v1.1.

Parameter	INCAGN02385 treatment group					
	25 mg Q2W (*n* = 4)	75 mg Q2W (*n* = 4)	250 mg Q2W (*n* = 4)	350 mg Q2W (*n* = 3)	750 mg Q2W (*n* = 7)	Total (*N* = 22)
Best overall response, *n* (%)						
Complete response	0	0	0	0	0	0
Partial response	0	0	0	0	0	0
Stable disease	2 (50)	0	1 (25)	1 (33)	2 (29)	6 (27)
> 6 months	1 (25)	0	1 (25)	0	2 (29)	4 (18)
Progressive disease	1 (25)	4 (100)	3 (75)	2 (67)	5 (71)	15 (68)
Not evaluable	1 (25)	0	0	0	0	1 (5)
ORR,^a^ % (95% CI)	0 (0-60)	0 (0-60)	0 (0-60)	0 (0-71)	0 (0-41)	0 (0-15)
DCR,^b^ % (95% CI)	50 (7-93)	0 (0-60)	25 (1-81)	33 (1-91)	29 (4-71)	27 (11-50)
PFS (months), median (95% CI)	9.3 (1.9-NE)	1.8 (1.7-NE)	2.2 (0.3-NE)	1.9 (1.9-NE)	1.9 (0.7-7.0)	1.9 (1.9-2.5)

^a^ORR was defined as the percentage of patients with complete or partial response by RECIST v1.1, as determined by investigator assessment.

^b^DCR was defined as the percentage of patients with complete or partial response as a best on-study response, or stable disease for ≥ 56 days by RECIST v1.1, as determined by investigator assessment.

Abbreviations: DCR, disease control rate; NE, not evaluable; ORR, objective response rate; PFS, progression-free survival; Q2W, every 2 weeks; RECIST, Response Evaluation Criteria in Solid Tumors.

## Discussion

This phase I open-label nonrandomized study assessed safety, tolerability, PK, pharmacodynamics, and preliminary activity of INCAGN02385 in 22 patients with select immunogenic solid tumors. No safety concerns were identified, and treatment was generally well tolerated in this heavily pretreated patient population in which > 60% of patients had received ≥ 3 lines of previous anticancer systemic therapy. Maximum tolerated dose was not reached at dose levels tested (up to 750 mg Q2W), and dose levels of 250 mg Q2W and above were determined to be PADs based on LAG-3 receptor occupancy and the T-cell proliferation profile. INCAGN02385 given at a dose level of 350 mg Q2W was therefore chosen for further study based on receptor occupancy, PK, pharmacodynamics, and safety data.

Overall tolerability was favorable across all dose levels, with only one patient discontinuing INCAGN02385, owing to a TEAE (transient ischemic attack, 350 mg Q2W dose group) that was not deemed treatment-related, and 6 patients (27%) requiring dose interruption owing to TEAEs, with one event deemed treatment-related. No grade 2-5 irAEs were reported. Two grade 1 irAEs (pneumonitis, peripheral, and sensory neuropathy) were reported per sponsor assessment; neither was considered related to INCAGN02385 treatment by investigators, and no dose-related TEAEs or trends suggestive of a drug effect were observed. No patients had infusion reactions, per investigator assessment. The safety profile of INCAGN02385 single-agent monotherapy is consistent with that observed for other single LAG-3 antibodies, ie, relatlimab,^25^ favezelimab,^18^ and ieramilimab,^19^ which are also reported to be associated with a low incidence of DLTs and with fatigue as the most common TRAE (27%, 20%, and 9% of patients, respectively).

The observation that INCAGN02385 doses ≥ 250 mg are associated with full soluble LAG-3 receptor occupancy at trough and increased CD4^+^ and CD8^+^ T-cell proliferation in some patients suggests that doses ≥ 250 mg may be sufficient to provide efficacy. However, as receptor occupancy was determined using an in vitro assay in isolated donor cells overexpressing LAG-3, a higher dose of INCAGN02385 (350 mg Q2W) was selected to account for potentially lower drug availability within the tumor.

INCAGN02385 PK parameters C_max_, AUC_0–t_, and AUC_0–∞_ were dose proportional across all dose levels evaluated. Although data were limited, an increasing trend was observed for INCAGN02385 trough serum concentrations from cycle 1 to cycle 7. In addition, while treatment-emergent ADAs were observed in 10/19 ADA-assessable patients overall, ADAs only impacted PK of 2 patients (both at the 25 mg Q2W dose level), and no treatment-emergent ADAs were seen at the 750 mg Q2W dose level.

Further studies are required to better understand the differential T-cell response seen at higher INCAGN02385 doses, including the increases in CD4^+^ T cells, and expansion of CD8^+^ T cells observed in some patients. There was no evidence of additional immune activation correlates. Elevation of IFNγ-inducible chemokines CXCL9 and CXCL10 is an established treatment effect with PD-1-targeted ICIs^26,27^; however, this was not observed following treatment with any dose level of INCAGN02385. Significant dose-independent elevation of soluble LAG-3 levels, observed following treatment with INCAGN02385, suggests a stabilizing effect of the antibody on the soluble protein. The possibility that increases in soluble LAG-3 levels could therefore be a pharmacodynamic marker for anti-LAG-3 treatment requires further validation.

No complete responses (CRs) or partial responses (PRs) to INCAGN02385 were observed in this heavily pretreated population. Six patients had SD with INCAGN02385 treatment; 5 of these patients had received previous treatment with a PD-1-targeted ICI (nivolumab or pembrolizumab), including one patient with hepatocellular carcinoma who had SD with combined PD-1 plus CTLA-4-targeted treatment (nivolumab plus ipilimumab). One patient with basosquamous carcinoma had previously demonstrated a PR with nivolumab, and the other 4 patients had progressed on previous ICI treatments. The present results are consistent with previous phase I/II studies of other anti–LAG-3 single-agent therapies,^18,19^ which reported that no patients receiving favezelimab^18^ or ieramilimab^19^ monotherapy had a CR or PR, and approximately 25% of patients receiving ieramilimab monotherapy had SD. In contrast, favezelimab and ieramilimab used in combination with anti–PD-1 therapy led to CRs or PRs in 5 patients (6%) and 13 patients (11%), respectively.^18,19^ Because of the overlapping mechanisms of action, a synergistic or additive effect is expected when INCAGN02385 is used in combination with anti–PD-1 therapy, and this has been reported in preclinical studies.^16^ Notably, a recent study among patients with advanced melanoma treated with cemiplimab (anti–PD-1)/fianlimab (anti–LAG-3) therapy showed increased response rates in PD-1/PD-L1 treatment-naive patients (*n* = 33) vs those with prior PD-1/PD-L1 treatment (*n *= 15) (ORR, 63.6%, CR, *n* = 3, PR, *n* = 18 vs ORR, 13.3%, CR, *n* = 1, PR, *n* = 1). This suggests that the benefits of treatment with anti–LAG-3 combination therapy may be more pronounced as first line.^28^

Limitations of the current study include the small number of patients per dose cohort, variability of the tumor histologies in treated patients, and the limited number of patients with positive immune stimulation markers. In view of the limited therapeutic efficacy with single agent LAG-3 antibodies seen in the current study and in other studies as described above, studies evaluating INCAGN02385 combination therapies with retifanlimab (anti–PD-1 mAb) and INCAGN02390 (anti–TIM-3 mAb) are underway and will help to elucidate the contribution of these antibodies to immunotherapy tumor response. In addition, alternative dosing schedules in doublet and triplet combinations continue to be explored.

## Conclusion

INCAGN02385 exhibited linear PK and preliminary evidence of disease control in this heavily pretreated patient population with advanced or malignant solid tumors. No DLTs were observed, and fatigue was the most commonly reported TRAE. The selected dose of INCAGN02385, 350 mg Q2W, is being explored in ICI combination studies. The efficacy and safety of INCAGN02385 in combination with retifanlimab and INCAGN02390 are currently being investigated in patients with select advanced malignancies (NCT04370704) and in patients with recurrent/metastatic squamous cell carcinoma of the head and neck (NCT05287113).

## Supplementary Material

oyaf136_suppl_Supplementary_Figures_S1-S3_Tables_S1-S5

## Data Availability

Incyte Corporation (Wilmington, DE, USA) is committed to data sharing that advances science and medicine while protecting patient privacy. Qualified external scientific researchers may request anonymized datasets owned by Incyte for the purpose of conducting legitimate scientific research. Researchers may request anonymized datasets from any interventional study (except phase I studies) for which the product and indication have been approved on or after January 1, 2020, in at least one major market (eg, US, EU, JPN). Data will be available for request after the primary publication or 2 years after the study has ended. Information on Incyte’s clinical trial data sharing policy and instructions for submitting clinical trial data requests are available at: https://www.incyte.com/Portals/0/Assets/Compliance%20and%20Transparency/clinical-trial-data-sharing.pdf?ver=2020-05-21-132838-960.
