# Supplementary material for: First-in-human phase I open-label study of the LAG-3 antagonist antibody INCAGN02385 in patients with select advanced or metastatic solid tumors
Source: Oncologist. 2025 Jul 9;30(7):oyaf136. doi: 10.1093/oncolo/oyaf136 (PMC12238943; doi:10.1093/oncolo/oyaf136)

**First-in-Human Phase I Open-Label Study of the LAG-3 Antagonist Antibody INCAGN02385 in Patients With Select Advanced or Metastatic Solid Tumors**

John D. Powderly II^1^, Martin E. Gutierrez^2^, Ani S. Balmanoukian^3^, Paul E. Hoyle^4^, Zhiwan Dong^4^, Lulu Cheng^4^, Xuejun Chen^4^, John E. Janik^4^, Nawel Bourayou^5^, Omid Hamid*^,3^

^1^Carolina BioOncology Institute, Huntersville, NC, USA

^2^Hackensack University Medical Center, Hackensack, NJ, USA

^3^The Angeles Clinic and Research Institute, a Cedars Sinai Affiliate, Los Angeles, CA, USA

^4^Incyte Corporation, Wilmington, DE, USA

^5^Incyte Biosciences International Sàrl, Morges, Switzerland

***Corresponding author:** Dr. Omid Hamid, The Angeles Clinic and Research Institute, a Cedars Sinai Affiliate, Los Angeles, CA, USA. Phone: (310) 294-0438. Email: [ohamid@theangelesclinic.org](mailto:ohamid@theangelesclinic.org)

**ORCID ID:** 0000-0002-8238-4655

**Table of** **Contents**

[SUPPLEMENTARY MATERIAL 3](#_Toc193791835)

[Supplementary Methods 3](#_Toc193791836)

[Eligible Tumor Types 3](#_Toc193791837)

[Rationale for Dose 3](#_Toc193791838)

[Dose Escalation Rules 4](#_Toc193791839)

[Criteria for Permanent Discontinuation of INCAGN02385 4](#_Toc193791840)

[Pharmacokinetic Analyses 5](#_Toc193791841)

[ADA Analyses 5](#_Toc193791842)

[LAG-3 Receptor Occupancy Analysis 6](#_Toc193791843)

[Supplementary Table S1. Definition of DLT (28-day DLT observation period). 7](#_Toc193791844)

[Supplementary Table S2. Treatment-related TEAEs occurring in >1 patient treated with INCAGN02385. 9](#_Toc193791845)

[Supplementary Table S3. Pharmacokinetic parameters of INCAGN02385 after first dose (cycle 1) and at steady state (cycle 6). 10](#_Toc193791846)

[Supplementary Table S4. Summary of immunogenicity for INCAGN02385. 13](#_Toc193791847)

[Supplementary Table S5. Select characteristics of patients achieving stable disease following treatment with INCAGN02385. 14](#_Toc193791848)

[Supplementary Figure S1. Patient disposition (*N* = 22). 15](#_Toc193791849)

[Supplementary Figure S2. INCAGN02385 dose proportionality after first dose. 16](#_Toc193791850)

[Supplementary Figure S3. Plasma protein analysis at cycle 1 day 1 (pre infusion), cycle 1 day 8, and cycle 2 day 1 in samples of patients treated with different doses of INCAGN02385. 17](#_Toc193791851)

## SUPPLEMENTARY MATERIAL

## Supplementary Methods

## Eligible Tumor Types

Eligible immunogenic tumor types included advanced or metastatic cervical cancer, microsatellite instability (MSI)–high endometrial cancer, gastric cancer (including stomach and gastroesophageal junction), esophageal cancer, hepatocellular carcinoma, melanoma (uveal melanoma excluded), Merkel cell carcinoma, mesothelioma, MSI-high colorectal cancer, non-small cell lung cancer, ovarian cancer, squamous cell carcinoma of the head and neck, small cell lung cancer, renal cell carcinoma, triple-negative breast cancer, and urothelial carcinoma, or alternative immunogenic tumor types with medical monitor approval.

## Rationale for Dose

The safe starting dose of 25 mg administered every 2 weeks (Q2W) was based on the average antibody concentration required for an individual to experience 50% of the maximum effect (EC_50_) [6.03 μg/mL] measured from 3 biologically relevant in vitro assays (ligand blockade [6.6 μg/mL], reporter cell line response [8.8 μg/mL], and cytokine secretion [2.7 μg/mL]) and converted to a projected dose of 31.5 mg (or 0.45 mg/kg in a 70 kg person) Q2W using a calculated average steady state serum concentration/dose of 13.3 from a 4-week toxicity study in cynomolgus monkeys (data on file, Incyte Corporation). The selected dose of 25 mg is 25-fold lower than the human equivalent dose for the no-observed-adverse-effect level in the 4-week cynomolgus monkey study (data on file, Incyte Corporation). Dose escalations were based on the need to distinguish PK parameters with a 3-fold increase in dose for each dose level increase. The maximum dose was based on the expectation that a 10 mg/kg dose level would be adequate to saturate receptor binding throughout the dosing interval.

## Dose Escalation Rules

The maximum tolerated dose (MTD) included defining the optimal dose administration schedule and the maximum number of tolerated doses. A minimum of 3 evaluable patients were enrolled in each cohort, commencing with cohort 1 (25-mg dose), and a 48-hour waiting period separated dose administration between the first and second patient of each dose cohort.

Dose-limiting toxicities (DLTs) were monitored during a 28-day observation period in the first set of patients enrolled within a cohort before enrollment of the next cohort (Supplementary Table S1). If 1 of the first set of evaluable patients enrolled had a DLT, then the cohort was expanded to include 3 additional evaluable patients. If no DLTs occurred in the first set of patients, or in the additional 3 patients if expanded, the dose was escalated. If a DLT occurred in at least one-third of the expanded cohort, then the MTD was considered exceeded, and the previous dose level deemed the MTD. If only 3 patients were treated at the MTD or pharmacologically active dose (PAD), then a minimum of 3 additional patients were enrolled before this dose was selected for further study. PAD was defined as a dose that reached a level of receptor occupancy and T-cell proliferation deemed to be biologically active for INCAGN02385. A dose that maintains >95% receptor occupancy would be required to achieve effective receptor occupancy for the LAG-3 antibody and therefore be deemed biologically active. An increase in Ki67 expression in CD4^+^ and/or CD8^+^ T cells was the anticipated biomarker to indicate reversal of T cell exhaustion with LAG-3 antibody administration.

## Criteria for Permanent Discontinuation of INCAGN02385

Patients experiencing unacceptable toxicity that was not caused by the underlying disease were permanently discontinued from the study. Unacceptable toxicity was defined as the occurrence of an adverse event related to INCAGN02385 that (in the opinion of the investigator or the sponsor’s medical monitor) compromises the ability of the patient to continue study-specific procedures or makes study continuation not in the patient’s best interest; or a persistent adverse event that requires a delay of INCAGN02385 therapy for >28 days without approval by the sponsor.

## Pharmacokinetic Analyses

Prepared standards, quality control samples, and patient serum samples diluted 1:500 in LowCross Buffer Strong (CANDOR Bioscience, Wangen, Germany) containing 1% Tween20, were added to a 96-well plate coated with the anti-INCAGN02385 antibody ABD29706. Bound INCAGN02385 was detected using the horseradish peroxidase (HRP)-conjugated detection antibody anti–immunoglobulin G Fc-HRP. Serum concentration data were analyzed using standard noncompartmental methods, performed using WinNonlin v8.3.2 (Certara USA Inc, Princeton, NJ, USA). An electrochemiluminescence immunoassay developed and validated by Frontage Laboratories (Exton, PA, USA) was used to detect, confirm, and titrate anti-INCAGN02385 antibodies in serum samples.

## ADA Analyses

Patient samples and controls were acidified by 1:5 dilution with acid treatment buffer (0.1 M glycine buffer, pH 3.0) and incubated at ambient temperature for 60 minutes, with shaking at 450 rpm. 100 µL of assay buffer (100 mM Tris, pH 8.0, 1% bovine serum albumin, 0.05% Tween20) followed by 50 µL of diluted controls and samples were added to an INCAGN02385-coated plate and incubated at 2-8°C for 16 hours, with shaking at 450 rpm. After plate washing, 150 µL of acid treatment buffer was added to each well to facilitate antidrug antibody (ADA) dissociation, and the plate was incubated at ambient temperature for 30 minutes, with shaking at 450 rpm. An MSD plate (Meso Scale Diagnostics, Rockville, MA, USA) was coated with 50 µL of dissociated ADA solution and incubated at ambient temperature for 1-2 hours, with shaking at 450 rpm. After plate washing, wells were blocked with 250 µL of casein in Tris buffered saline (TBS; Thermo Fisher Scientific, Waltham, MA, USA) per well and the plate incubated at ambient temperature for 60 minutes, with shaking at 450 rpm. For screening and titration assays: after plate washing, 100 µL of detection solution (casein in TBS, Thermo Fisher Scientific) with 0.5 µg/mL ruthenylated INCAGN02385 (Frontage Laboratories) was added to each well and the plate was incubated at ambient temperature for 50-60 minutes, with shaking at 450 rpm in the dark. For confirmation assays: after plate washing, 100 µL of detection solution with 0.5 µg/mL ruthenylated INCAGN02385 and 5 µg/mL INCAGN02385 was added to each well and the plate was incubated at ambient temperature for 50-60 minutes, with shaking at 450 rpm in the dark. After washing, 100 µL of 2× read buffer was added to each well and the plate immediately read on an MSD Sector Imager S600 (Meso Scale Diagnostics, Rockville, MA, USA) to determine the presence of ADAs. Samples were classified as ADA positive if percent inhibition of the inhibited sample was ≥30% (confirmation cut point).

## LAG-3 Receptor Occupancy Analysis

Whole blood samples from healthy volunteers were incubated with staphylococcal enterotoxin B (48 hours at 37°C, 5% CO_2_) to increase T-cell lymphocyte-activation gene 3 (LAG-3) expression. T cells were then incubated with patient serum samples for 30 minutes. Following incubation, washed cells were stained with competing (anti–LAG-3 clone 7H2C65, conjugated with phycoerythrin) and noncompeting (anti–LAG-3 clone T47-530, conjugated with allophycocyanin) antibodies, and analyzed by flow cytometry.

## Supplementary Table S1. Definition of DLT (28-day DLT observation period).

| **Nonhematologic toxicity** |
| --- |
| - Any liver function abnormalities that meet the definition of Hy’s law^a^ - Encephalopathy of any grade - Any grade ≥3 nonhematologic toxicity except the following: - Transient (≤72 hours) abnormal laboratory values without associated clinically significant signs or symptoms - Nausea, vomiting, and diarrhea adequately controlled with supportive care within 48 hours - Changes in cholesterol and triglycerides - An event clearly associated with the underlying disease, disease progression, a concomitant medication, or comorbidity - Asymptomatic changes in lipid profiles - Asymptomatic changes in amylase and lipase - Singular or nonfasting elevations in blood glucose (ie, blood glucose excursions will be considered toxicities if fasting blood glucose is elevated on 2 separate occasions) |
| **Hematologic toxicity** |
| - Grade 3 thrombocytopenia with clinically significant bleeding (ie, requires hospitalization, transfusion of blood products, or other urgent medical intervention) - Grade 4 thrombocytopenia - Grade ≥3 febrile neutropenia (absolute neutrophil count <1.0 × 10^9^/L and fever >101°F/38.3°C) - Grade 4 neutropenia that does not recover to grade ≤2 in ≤3 days after interrupting study drug - Grade 4 anemia not explained by underlying disease or some other concomitant disorder |
| **Immune-related toxicity** |
| - Grade ≥2 ocular irAEs - Grade 3 irAEs that do not improve to baseline or grade ≥1 in <5 days with appropriate care or with corticosteroid therapy - Grade 4 irAEs, regardless of duration |
| **General** |
| - Inability to receive the planned number of doses within the 28-day DLT period due to toxicity, regardless of grade |
| **Maximum tolerated dose** |
| - MTD will be defined as 1 dose level below that at which at least one-third of patients in a particular cohort have DLTs |
| **Maximum number of tolerated doses** |
| - For each dose and schedule explored, if >33% of patients (minimum of 6 patients) experience a grade ≥3 toxicity related to study drug after completing ≥4 cycles, the sponsor and investigators will discuss possible MNTD and actions to be taken based on all available safety data. All AEs, regardless of the time of occurrence on study, may be considered in DLT determination decisions |

^a^Hy's law is defined as (1) ALT or AST elevation >3 × ULN, (2) total bilirubin >2 × ULN without initial findings of cholestasis (elevated serum alkaline phosphatase), and (3) no other apparent possible causes of aminotransferase elevation and hyperbilirubinemia, including, but not limited to, viral hepatitis, pre-existing chronic or acute liver disease, or the administration of other drug(s) known to be hepatotoxic.
Abbreviations: AE, adverse event; ALT, alanine aminotransferase; AST, aspartate aminotransferase; DLT, dose-limiting toxicity; irAE, immune-related adverse event; MNTD, maximum number of tolerated doses; MTD, maximum tolerated dose; ULN, upper limit of normal.

## **Supplementary Table S2.** Treatment-related TEAEs occurring in >1 patient treated with INCAGN02385.

| **MedDRA preferred term,  *n* (%)** | **INCAGN02385 treatment group** | | | | | |
| --- | --- | --- | --- | --- | --- | --- |
|  | **25 mg Q2W (*n* = 4)** | **75 mg Q2W  (*n* = 4)** | **250 mg Q2W (*n* = 4)** | **350 mg Q2W (*n* = 3)** | **750 mg Q2W  (*n* = 7)** | **Total (*N* = 22)** |
| Any treatment-related TEAE | 3 (75) | 3 (75) | 2 (50) | 3 (100) | 5 (71) | 16 (73) |
| Fatigue | 2 (50) | 0 | 0 | 3 (100) | 2 (29) | 7 (32) |
| Blood creatinine increased | 0 | 0 | 1 (25) | 0 | 1 (14) | 2 (9) |
| Lymphopenia | 0 | 1 (25) | 0 | 1 (33) | 0 | 2 (9) |
| Myalgia | 0 | 0 | 1 (25) | 0 | 1 (14) | 2 (9) |
| Pruritus | 1 (25) | 0 | 0 | 0 | 1 (14) | 2 (9) |
| Tumor pain | 1 (25) | 1 (25) | 0 | 0 | 0 | 2 (9) |

Abbreviations: MedDRA, Medical Dictionary for Regulatory Activities; Q2W, every 2 weeks; TEAE, treatment-emergent adverse event.

## Supplementary Table S3. Pharmacokinetic parameters of INCAGN02385 after first dose (cycle 1) and at steady state (cycle 6).

| **INCAGN02385 treatment group** | **C_max_**  **(mg/L)** | **t_max_**  **(hours)** | **AUC_0–t_**  **(mg·h/L)** | **AUC_0–∞_**  **(mg·h/L)** | **t_½_**  **(hours)** | **CL**  **(L/h)** | **V_z_**  **(L)** |
| --- | --- | --- | --- | --- | --- | --- | --- |
| Pharmacokinetic parameters of INCAGN02385 after first dose | | | | | | | |
| 25 mg Q2W  (*n* = 4^a^) | 7.9 ± 3.0  (7.3) | 4.3  (0.8-28) | 1170 ± 792  (935) | 2490 | 131 | 0.0101 | 1.9 |
| 75 mg Q2W  (*n* = 4) | 22.3 ± 13.3  (19.8) | 2.3  (0.50-4.1) | 2690 ± 787  (2600) | 3630 ± 1070  (3510) | 159 ± 24.9  (158) | 0.0222 ± 0.00716  (0.0214) | 4.9 ± 1.0  (4.9) |
| 250 mg Q2W (*n* = 2) | 104, 78.8 | 0.5, 0.6 | 13 300, 12 800 | 22 300, 19 800 | 266, 211 | 0.0112, 0.0126 | 4.3, 3.9 |
| 350 mg Q2W (*n* = 3) | 95.1 ± 35.1  (91.0) | 4.1  (0.5-4.7) | 13 800 ± 2240  (13 700) | 19 400 ± 4060  (19 100) | 174 ± 30.5  (173) | 0.0186 ± 0.00442  (0.0183) | 4.6 ± 0.9  (4.6) |
| 750 mg Q2W (*n* = 6) | 434 ± 501  (308) | 0.625  (0.5-4.1) | 37 400 ± 4710  (37 100) | 60 300 ±  10 900  (59 400) | 234 ± 43.2  (230) | 0.0128 ± 0.00273  (0.0126) | 4.2 ± 0. 6  (4.2) |
| Pharmacokinetic parameters of INCAGN02385 at steady state | | | | | | | |
| 25 mg Q2W  (*n* = 1^b^) | 7.56 | 4.3 | 1560 | — | — | — | — |
| 75 mg Q2W  (*n* = 2) | 30.6, 44.0 | 0.7, 0.7 | 5630, 5420 | — | 277, 513 | 0.00805, 0.00622 | 3.2, 4.6 |
| 250 mg Q2W (*n* = 1) | 129 | 4.5 | 25 400 | — | 279 | 0.00533 | 2.1 |
| 350 mg Q2W (*n* = 2) | 147, 123 | 4.1, 0.6 | 31 300, 27 700 | — | 121, 349 | 0.0104, 0.00622 | 1.8, 3.1 |
| 750 mg Q2W (*n* = 2) | 489, 341 | 0.6, 0.7 | 99 500, 71 800 | — | 376, 502 | 0.00331, 0.00398 | 1.8, 2.9 |

Values are mean ± standard deviation (geometric mean) except t_max_, which is median (minimum, maximum). Individual values are provided when *n* < 3 patients.
^a^AUC_0–∞_, t_½_, CL, and V_z_ were only determined in 1 patient. ^b^t_½_, CL, and V_z_ were not determined in the patient.
Abbreviations: AUC_0–∞_, area under the serum concentration–time curve from time 0 to infinity; AUC_0–t_, area under the serum concentration–time curve from time 0 to the last measurable concentration at time t; CL, clearance; C_max_, maximum observed serum concentration; Q2W, every 2 weeks; t_½_, apparent terminal-phase disposition half-life; t_max_, time to maximum concentration; V_z_, volume of distribution during the elimination phase.

## Supplementary Table S4. Summary of immunogenicity for INCAGN02385.

| **INCAGN02385  treatment group** | **Total number of patients with  ADA-assessable samples**^a^ | **Patients**  **with treatment-emergent**  **positive immunogenicity status,^b^ *n* (%)** |
| --- | --- | --- |
| 25 mg Q2W | 4 | 3 (75) |
| 75 mg Q2W | 4 | 3 (75) |
| 250 mg Q2W | 2 | 2 (100) |
| 350 mg Q2W | 3 | 2 (67) |
| 750 mg Q2W | 6 | 0 |
| All treatment groups | 19 | 10 (53) |

^a^ADA-assessable patients were defined as those with ≥1 postdose sample with a reportable ADA result, either negative or positive. ^b^Treatment-emergent ADA positive refers to patients who were negative at baseline but had ≥1 positive ADA sample post baseline.

Abbreviations: ADA, antidrug antibody; Q2W, every 2 weeks.

## **Supplementary Table S5.** Select characteristics of patients achieving stable disease following treatment with INCAGN02385.

| Parameter | Patient 1 | Patient 2 | Patient 3 | Patient 4 | Patient 5 | Patient 6 |
| --- | --- | --- | --- | --- | --- | --- |
| INCAGN02385 treatment group | 25 mg Q2W | 25 mg Q2W | 250 mg Q2W | 350 mg Q2W | 750 mg Q2W | 750 mg Q2W |
| Tumor type | Lung | Salivary gland | Hepatocellular carcinoma | Gastric | Adenocarcinoma of endometrium | Basosquamous carcinoma |
| Prior systemic therapy | Chemotherapy, targeted therapy, ICI (nivolumab), other immunotherapy (OX40). | Chemotherapy, targeted therapy, ICI (nivolumab). | Targeted therapy, ICI (nivolumab + ipilimumab). | Chemotherapy | Chemotherapy, hormonal therapy, ICI (pembrolizumab). | Chemotherapy, targeted therapy, ICI (nivolumab). |
| Prior radiotherapy | N | Y | N | N | Y | Y |
| Prior surgery | N | Y | Y | Y | Y | Y |
| History of PD on prior treatment | Y | Y | Y | Y | Y | Y |
| PFS duration^a^ (months) | 9.3 | 1.9 | 7.3 | 5.3 | 7.0 | 13.7 |

^a^Associated with BOR of stable disease.

Abbreviations: BOR, best overall response; ICI, immune checkpoint inhibitor; PD, progressive disease; Q2W, every 2 weeks.

Supplementary Figure S1. Patient disposition (*N* = 22). ^a^Patient had another complicating disease (chronic lymphocytic leukemia). Q2W, every 2 weeks.

**
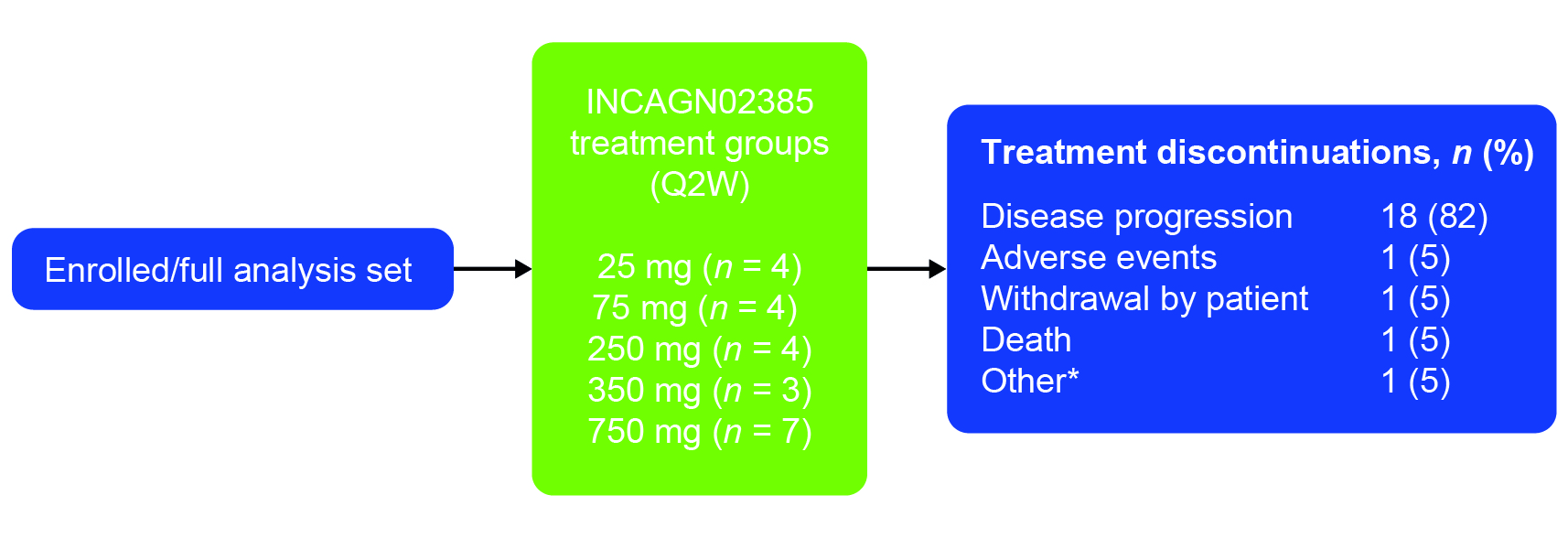
**

Supplementary Figure S2. INCAGN02385 dose proportionality after first dose. INCAGN02385 dose level (range, 25-750 mg) versus serum PK parameter (A) C_max_, (B) AUC_0–t_, and (C) AUC_0–∞_ values, respectively. Scatterplots depict individual patients across all treatment groups and include the regression line (black) and 90% confidence intervals (red). AUC_0–∞_, area under the serum concentration–time curve from time 0 to infinity; AUC_0–t_, area under the serum concentration–time curve from time 0 to the last measurable concentration at time t; C_max_, maximum observed serum concentration; PK, pharmacokinetic; Q2W, every 2 weeks.


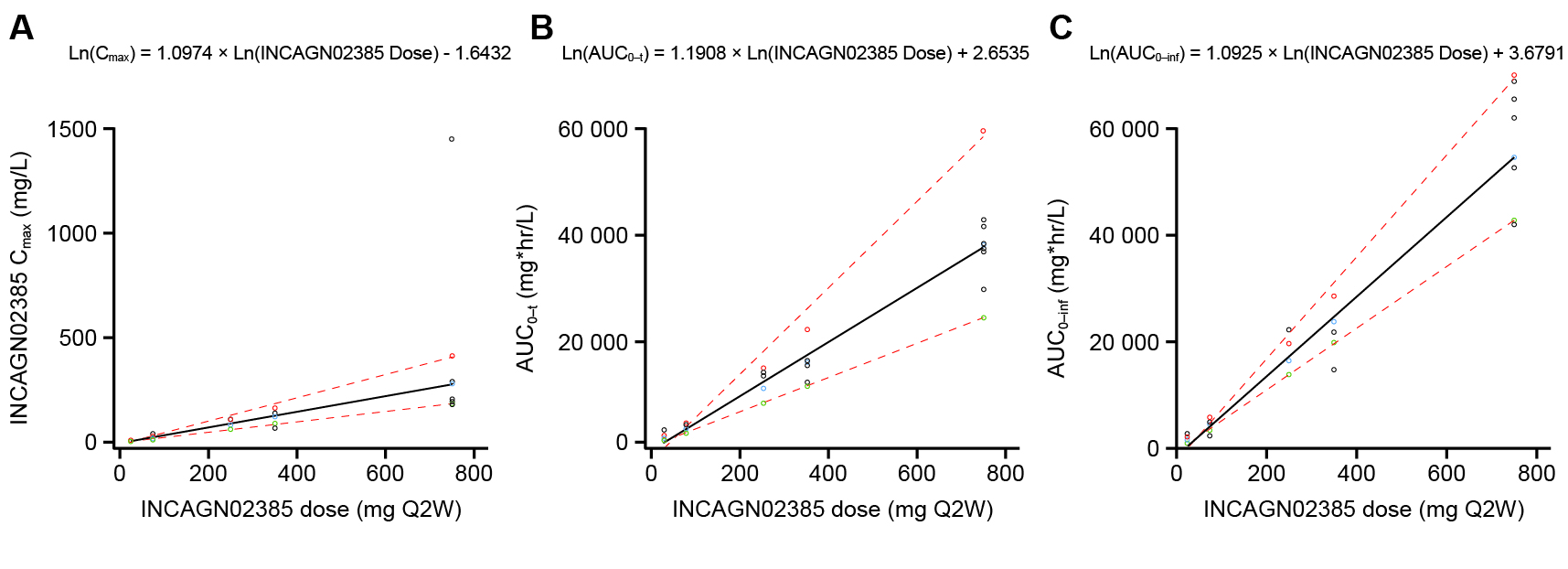


Supplementary Figure S3. Plasma protein analysis at cycle 1 day 1 (pre infusion), cycle 1 day 8, and cycle 2 day 1 in samples of patients treated with different doses of INCAGN02385. Each individual patient is depicted by a single line. (A) Line graph shows levels of soluble LAG-3. (B) Line graph shows levels of soluble CXCL-9, CXCL-10, and CXCL-11. C, cycle; D, day; LAG3, lymphocyte-activation gene 3; Q2W, every 2 weeks.


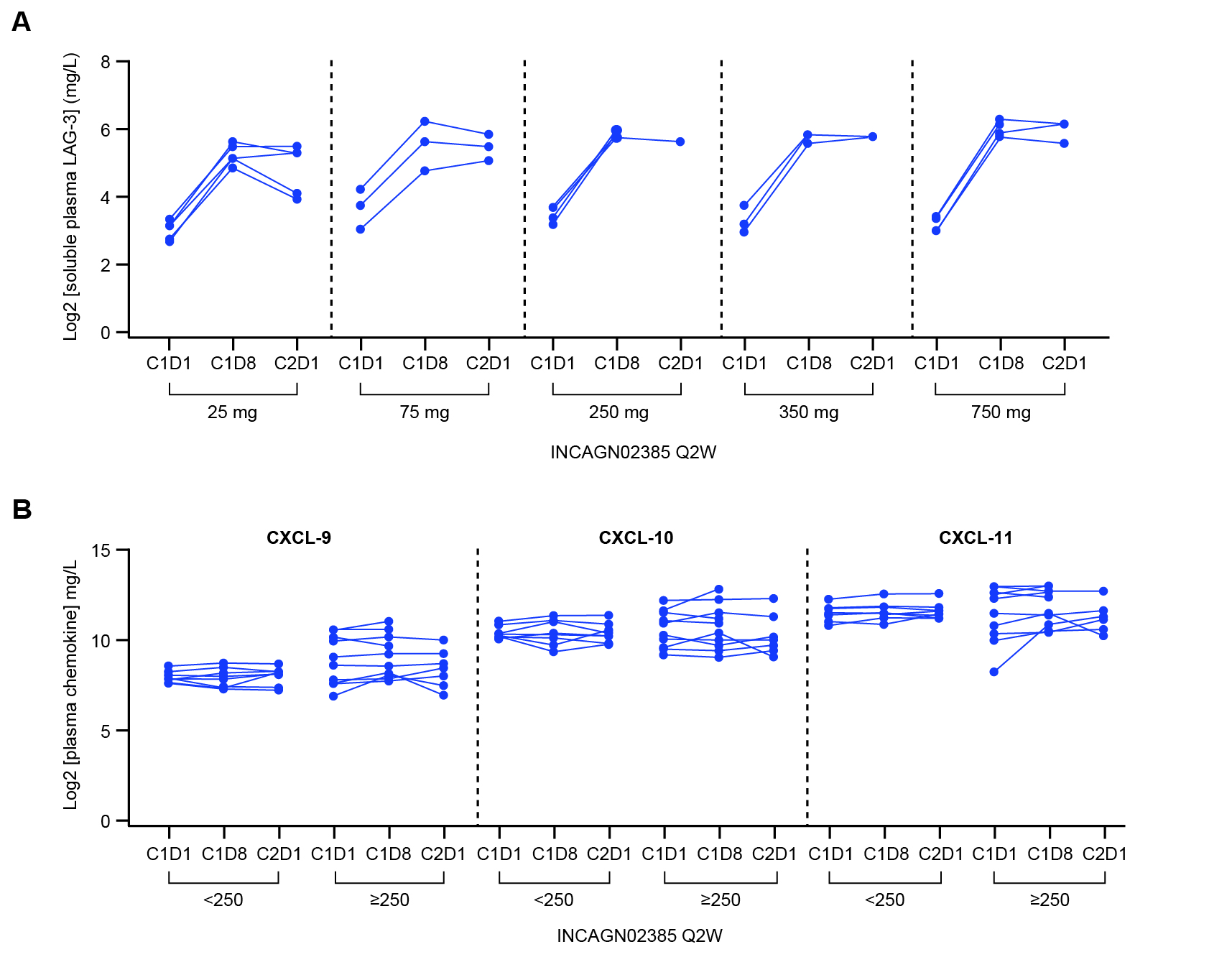

Supplement: oyaf136_suppl_Supplementary_Figures_S1-S3_Tables_S1-S5 [file oyaf136_suppl_supplementary_figures_s1-s3_tables_s1-s5.docx]
